# Soil aggregate size influences the impact of inorganic nitrogen deposition on soil nitrification in an alpine meadow of the Qinghai–Tibet Plateau

**DOI:** 10.7717/peerj.8230

**Published:** 2020-01-07

**Authors:** Jingjing Li, Chao Yang, Xiaoli Liu, Hanzhong Ji, Xinqing Shao

**Affiliations:** 1 College of Grassland Science and Technology, China Agricultural University, Beijing, China; 2 Grassland Agri-Husbandry Research Center, College of Grassland Science, Qingdao Agricultural University, Qingdao, China; 3 Institute of Haibei Tibetan Autonomous Prefecture Animal Husbandry and Veterinary Science, Xining, China; 4 Technical Platform for Adaptive Management of Livestock System in Alpine Grassland, Xining, China; 5 Key Laboratory of Restoration Ecology of Cold Area in Qinghai Province, Northwest Institute of Plateau Biology, Chinese Academy of Sciences, Xining, China

**Keywords:** Soil aggregate, Alpine meadow, Soil nitrification, Inorganic nitrogen deposition, Soil porosity

## Abstract

**Background:**

Ammonium (NH_4_^+^) and nitrate (NO_3_^−^) are two inorganic forms of nitrogen (N) that are deposited from the atmosphere into soil systems. As the substrate and product of soil nitrification, these two forms of inorganic nitrogen will affect or be affected by the soil net nitrification rate (N_r_). Our knowledge regarding soil nitrification is mainly derived from studies with bulk soil. However, soil is composed of different aggregate fractions, which may have an important impact on N_r_.

**Methods:**

In 2017, we collected soil samples from an alpine meadow of the Qinghai–Tibet Plateau and separated them into four soil aggregates (2–4, 1–2, 0.25–1, and <0.25 mm) using the dry sieving method. The four soil aggregate sizes amended with the 2 N deposition forms (NH_4_^+^-N and NO_3_^−^-N) were then incubated at 25 °C for 28 days, and the soil aggregates for each treatment were collected on day 0, 7, 14, 21, and 28 to determine the NO_3_^−^-N concentration. The soil N_r_ and contribution of soil aggregates to the nitrification rate in the bulk soil were calculated.

**Results:**

There were differences in the physicochemical properties of the soil aggregates. The addition of N and aggregate size had strong effects on soil N_r_, which were significantly increased under high levels of NH_4_^+^ addition across all soil aggregates. The N_r_ during the 4 week incubation period differed among aggregate sizes. N_r_ in the 2–4 mm aggregates was higher than in the other aggregates, which was correlated with the maximum values of the soil porosity observed in the 2–4 mm aggregates. Furthermore, almost half of the soil was composed of aggregates of <0.25 mm, indicating that the <0.25 mm aggregates made a higher contribution to the nitrification rate in the bulk soil than the other aggregates, even though these aggregates had a lower nitrification ability. Overall, our study revealed that the soil nitrification rate was influenced by both the N addition and soil aggregates, and that the 2–4 mm aggregates had a dominant effect on the response of soil N transformation processes to future nitrogen deposition in the alpine meadow.

## Introduction

Global nitrogen (N) deposition has increased continuously and is a large N source for many terrestrial ecosystems (Goodale, Dise & Sutton, 2011). Most studies conducted to date have primarily focused on the atmospheric N that enters the soil as the wet deposition of inorganic N (Hao et al., 2017; Liu et al., 2015), which mainly occurs in the chemical forms of ammonium (NH_4_^+^) and nitrate (NO_3_^−^) (Yang et al., 2010; Zhao et al., 2009). In addition, the NH_4_^+^/NO_3_^−^ ratio in wet deposition decreased from 1980s, and the contribution from NO_3_^−^ has been increasingly important in the total N deposition (Zhao et al., 2009). Nitrification is an important N transformation process, in which gaseous N_2_O and NO_3_^−^ (Li et al., 2015b; Zhao et al., 2017) are produced as intermediate or end products (Zhao, Cai & Xu, 2007). NH_4_^+^ is considered to stimulate nitrification via increasing the substrate (Zhao, Cai & Xu, 2007), but as the end product of nitrification, the effects of NO_3_^−^ have rarely been considered (Ying et al., 2017). The excessive concentration of the final product (NO_3_^−^) might inhibit the activity of nitrosating bacteria and nitrifying bacteria, thereby affecting soil nitrification capacity (Painter, 1977). Net nitrification rate (N_r_) is generally driven by multiple soil factors, including soil pH (Xiao, Schaefer & Yang, 2017), organic matter (Figueiredo, Enrich-Prast & Rütting, 2016), temperature and moisture (Ma et al., 2017a), land use (Li et al., 2015a), and microbial activity (Li et al., 2018).

Soil aggregates, which are the soil particles combined with organic and inorganic matter (Six, Elliott & Paustian, 2000), are conventionally divided into macro-aggregates (>0.25 mm) and micro-aggregates (<0.25 mm) (Tisdall & Oades, 1982). Most previous studies of soil aggregates have focused on carbon sequestration or mineralization in areas that have experienced land use changes (Rabbi et al., 2015) and areas in tillage systems (Xie et al., 2017), while the research regarding nitrogen-related processes, such as nitrification, is insufficient (Han et al., 2019; Hoffmann, Schloter & Wilke, 2007; Nishio & Furusaka, 1970). Jiang et al. (2015) separated the soil into three aggregate fractions, including large macroaggregates (>2 mm), small macroaggregates (0.25–2 mm) and inter-aggregate soil and space (<0.25 mm), and found soil aggregates showed a remarkable effect on potential nitrification activity and ammonia oxidizers in an acidic soil. Aggregates not only physically protect soil organic carbon (Six, Elliott & Paustian, 2000), but also limit oxygen diffusion (Khalil, Mary & Renault, 2004; Sexstone et al., 1985) and determine nutrient adsorption (Wang, Yost & Linquist, 2001). All of these processes have profound effects on N_r_. The macropores of soil aggregates, with low tortuosity and high pore connectivity, result in highly variable flows of gas and water (Jarvis, 2007). Oxygen availability varying along the aggregate radius is the main environmental factor influencing the N transformation (Kremen et al., 2005). A research found that due to limited oxygen supply, anaerobic conditions become prevalent with increasing soil aggregate size (5 mm, 10 mm, 15 mm, 20 mm), and nitrification occurs in the aerobic part of the aggregates, close to its surface (Kremen et al., 2005). Given the importance of the physical structure of soil aggregates, it is important to investigate how soil aggregates affect nitrification in an alpine meadow of the Qinghai–Tibet Plateau under N deposition. The effects of soil aggregate size on N_r_ have not been thoroughly investigated in previous studies, especially in alpine meadows of the Qinghai–Tibet Plateau, where N deposition has significantly increased (Liu et al., 2013).

The Qinghai–Tibet plateau, which is the highest and largest plateau on Earth, is experiencing a sharp increase in N deposition and changes in precipitation (Xiong et al., 2016). Alpine meadows, which occupy approximately 35% of the plateau, comprise one of the most important ecological types on the Qinghai–Tibet Plateau. The rate of N deposition has increased significantly, reaching 13.8 kg ha^−1^ year^−1^ in the eastern Qinghai–Tibet Plateau during the period 1980–2010 (Liu et al., 2013). In the present study, an incubation experiment was conducted with two forms of N addition (NH_4_^+^-N and NO_3_^−^-N) and four soil aggregate fractions (2–4, 1–2, 0.25–1 and <0.25 mm). The NO_3_^−^-N concentrations were determined on day 0, 7, 14, 21 and 28, and the soil N_r_ was calculated during the 4 weeks. Our specific goals were to (1) understand the effects of different aggregate sizes on the N_r_ and (2) analyze the effects of different N forms on N_r_ among different aggregate size fractions. We hypothesized that the macro-aggregates have a higher N_r_ than the micro-aggregates among all nitrogen addition treatments might due to the greater soil porosity (SP). We also expected to obtain information regarding how N_r_ in soil aggregates contributes to the overall N_r_ in the bulk soil.

## Materials and Methods

### Soil sampling and sieving of aggregates

The soil was collected in an alpine meadow located at the Haibei Demonstration Zone of the Plateau Modern Ecological Animal Husbandry Science and Technology (36°55′N, 100°57′E) in Qinghai Province, China, at an altitude of 3,040 m. The mean annual temperature and precipitation of this site are −0.45 °C and 400 mm, respectively. The minimum monthly mean air temperature is −29 °C in January, with the maximum of 27 °C occurring in July. The dominant plant species belong to the Gramineae family and include *Elymus dahuricus* and *Stipa capillata*. The soil underlying the site is a clay–loam classified as Mat–Gryic Cambisol (Ma et al., 2017b).

In June of 2017, soil cores (0–15 cm) were collected at random from a natural alpine meadow with an area of about 50 × 50 m that was not subject to any management or use practices. We set up three sampling points along the diagonal of the selected area as three replicates of field soil sampling. At each sampling point, we randomly collected soil cores (5 cm in diameter) and took approximately 50 kg of bulk soil. We then transported samples to the laboratory where plant roots and fine stones were carefully removed by hand and soils were sieved to the different soil aggregate sizes required for the experiment. The three field sampling points corresponded to the three replicates in the laboratory. Four aggregate-size classes were obtained by dry sieving 100 g of fresh soil through a series of four sieves (4, 2, 1, and 0.25 mm) as follows: large macro-aggregates (2–4 mm), macro-aggregates (1–2 mm), meso-aggregates (0.25–1 mm), and micro-aggregates (<0.25 mm). Soil was placed on a four mm sieve, then manually moved up and down by 10 cm 60 times during a period of 2 min. The material passing through the four mm sieve was then transferred to the next smaller-sized sieve (two mm) for further fractionation, ultimately generating four aggregate fractions (Jiang et al., 2015; Yang, Liu & Zhang, 2017). This process was repeated until the amount of each soil aggregate size fraction required was obtained.

### Determination of physicochemical properties for soil aggregates

Soil organic carbon (SOC) was determined using an auto-analyzer (TOC, Elementar, Germany). Ten gram of fresh soil was extracted with 50 ml of 2 M KCl to measure soil NH_4_^+^-N and NO_3_^−^-N using a flow-solution analyzer (Flowsys, Ecotech, Germany). Soil pH was measured using a pH meter after shaking a 1:2.5 air-dried soil/water suspension for 30 min. SP was calculated from the bulk density (BD) and the particle density (2.65 g cm^−3^) using the following equation (Munkholm et al., 2016):

$${\rm Soil\; porosity} = \left( {1 - {\rm Soil\; bulk\; density}/2.65} \right) \times 100{\rm \% }$$

Soil BD was determined using oven-dried soils (Regelink et al., 2015). Briefly, we placed three replicates of 500 g aggregates in 1,000 ml jars. We then adjusted the moisture content to 30%, which was the maximum field water capacity of the soil. After allowing the samples to settle for 1 day, a foil sampler with a volume of 100 cm^3^ was used to obtain the samples. This was followed by drying at 105 °C for 1 day.

### N addition and soil aggregate incubation

Soil aggregate samples (200 g) of four classes were placed at the bottom of 1,000 ml plastic bottles. Polyethylene film punctured with needle holes was then placed on all bottles to maintain aerobic conditions. The soil moisture content was adjusted to 60% field moisture capacity, and the bottles were pre-incubated at 25 °C for 7 days. Our experiment employed a two-factor design that consisted of four levels of aggregate sizes (2–4, 1–2, 0.25–1, and <0.25 mm) and two forms of nitrogen (N) addition (i.e., NH_4_^+^-N and NO_3_^−^-N). Two forms of N were applied as NH_4_Cl and Ca (NO_3_)_2_, which were added to the four levels of soil aggregates to give a gradient of 0, 5, and 10 mg N kg^−1^ soil. Each treatment had three replicates. After incubation for 0, 7, 14, 21 and 28 days, 10 g of wet soil was collected from three replicate bottles of each treatment. The NO_3_^−^-N was then extracted with 50 ml of 2 M KCl, after which the filtrate was used to determine the NO_3_^−^-N concentrations.

### Data calculations and analysis

The net nitrification rate N_r_ (mg NO_3_^−^-N kg^−1^ aggregate day^−1^) was calculated from the equation below (Xin et al., 2014):

$${N_{\rm{r}}} = \left[ {{{\left( {{\rm{NO}}_3^ - {\text{-}}{\rm{ N}}} \right)}_a} - {{\left( {{\rm{NO}}_3^ - {\text{-}}{\rm{ N}}} \right)}_b}} \right]/{T_{\rm{d}}}$$

where *a* and *b* are the NO_3_^−^-N concentrations measured after and before each incubation period, respectively, and *T*_d_ indicates the incubation time in days.

The contribution of each type of soil aggregates to the net nitrification rate of bulk soil was determined from the following equation (Yang, Liu & Zhang, 2017):

$${{\rm C}_{\rm r}} = {{\rm N}_{\rm r}} \times {{\rm A}_{\rm r}}$$

where C_r_ is the contribution rate (mg NO_3_^−^-N kg^−1^ soil day^−1^), N_r_ is the nitrification rate observed during the fourth week and A_r_ is the aggregates proportion (%).

The homogeneity and normality of variances were verified for all data using the Levene and Kolmogorov–Smirnov tests, respectively. Repeated-measures analysis of variance (ANOVA) was employed to test the effects of the incubation time, soil aggregates, and N addition on N_r_. One-way ANOVA was used to test the physicochemical properties among soil aggregate sizes and the differences of NO_3_^−^-N concentrations, N_r_ and C_r_ among soil aggregate sizes or the differences of NO_3_^−^-N concentrations, N_r_ and C_r_ under nitrogen addition treatment with different concentrations and forms. Following ANOVA, post hoc comparisons of the means were calculated using Tukey multiple comparison (*P* < 0.05). Pearson’s correlation was used to determine the correlation between soil physicochemical properties and N_r_ for all nitrogen addition treatments. All statistical analyses were performed using the SPSS statistical package (version 19.0; IBM, Armonk, NY, USA) and figures were obtained using SigmaPlot 12.5.

## Results

### Physicochemical properties of soil aggregates

The SOC, NH_4_^+^-N, and NO_3_^−^-N concentrations were significantly different among the aggregate sizes (Table 1). The level of SOC associated with the <0.25 mm aggregates was significantly higher than 2–4 and 0.25–1 mm soil aggregates. The NH_4_^+^-N concentration in the 2–4 mm aggregates was significantly higher than in the other soil aggregates sizes, which did not significantly differ in their concentration. The NH_4_^+^-N concentration in the 2–4 mm aggregates was 14.1% higher than that in the 0.25–1 mm aggregates. The NO_3_^−^-N concentrations in the 2–4 mm and 1–2 mm aggregates were significantly higher than in the 0.25–1 mm and <0.25 mm aggregates. The soil pH was alkaline for all the soil aggregates sizes, varying between 8.67 and 8.93. Soil pH was significantly lower in 1–2 mm and <0.25 mm aggregates than in aggregates with other sizes. The BD of <0.25 and 0.25–1 mm aggregates were significantly higher than for the other soil aggregates sizes, and the BD of the 2–4 mm aggregate fraction was the lowest (0.78 g cm^−3^; which was 28.4% lower than the BD of <0.25 mm aggregates). The 2–4 mm aggregates had the highest porosity (70.48%), which was significantly higher than in the other soil aggregate sizes.

**Table 1:** Physiochemical properties (means ± SE, *n* = 3) of different soil aggregates.

Aggregates	SOC (g kg^−1^)	NH_4_^+^-N (mg kg^−1^)	NO_3_^−^-N (mg kg^−1^)	Soil pH	BD (g cm^−3^)	SP (%)	Aggregate proportion (%)
2–4 mm	7.49 ± 0.14b	8.09 ± 0.14a	3.63 ± 0.28a	8.85 ± 0.03a	0.78 ± 0.01c	70.48 ± 0.40a	10.48 ± 0.62c
1–2 mm	8.47 ± 0.13a	7.54 ± 0.14b	3.54 ± 0.35a	8.67 ± 0.01b	0.99 ± 0.01b	62.42 ± 0.24b	12.21 ± 0.50c
0.25–1 mm	6.65 ± 0.23c	7.09 ± 0.04b	0.87 ± 0.22b	8.93 ± 0.03a	1.06 ± 0.01a	59.84 ± 0.37c	28.35 ± 1.09b
<0.25 mm	8.59 ± 0.08a	7.27 ± 0.18b	0.52 ± 0.03b	8.71 ± 0.05b	1.09 ± 0.01a	59.01 ± 0.44c	48.73 ± 1.83a

**Note:**

SOC, soil organic carbon; BD, bulk density; SP, soil porosity. Different letters in the columns represent significant differences between the soil aggregate sizes (*P* < 0.05).

### Response of NO_3_^−^-N concentration to soil aggregate sizes and N addition

**Figure 1:**
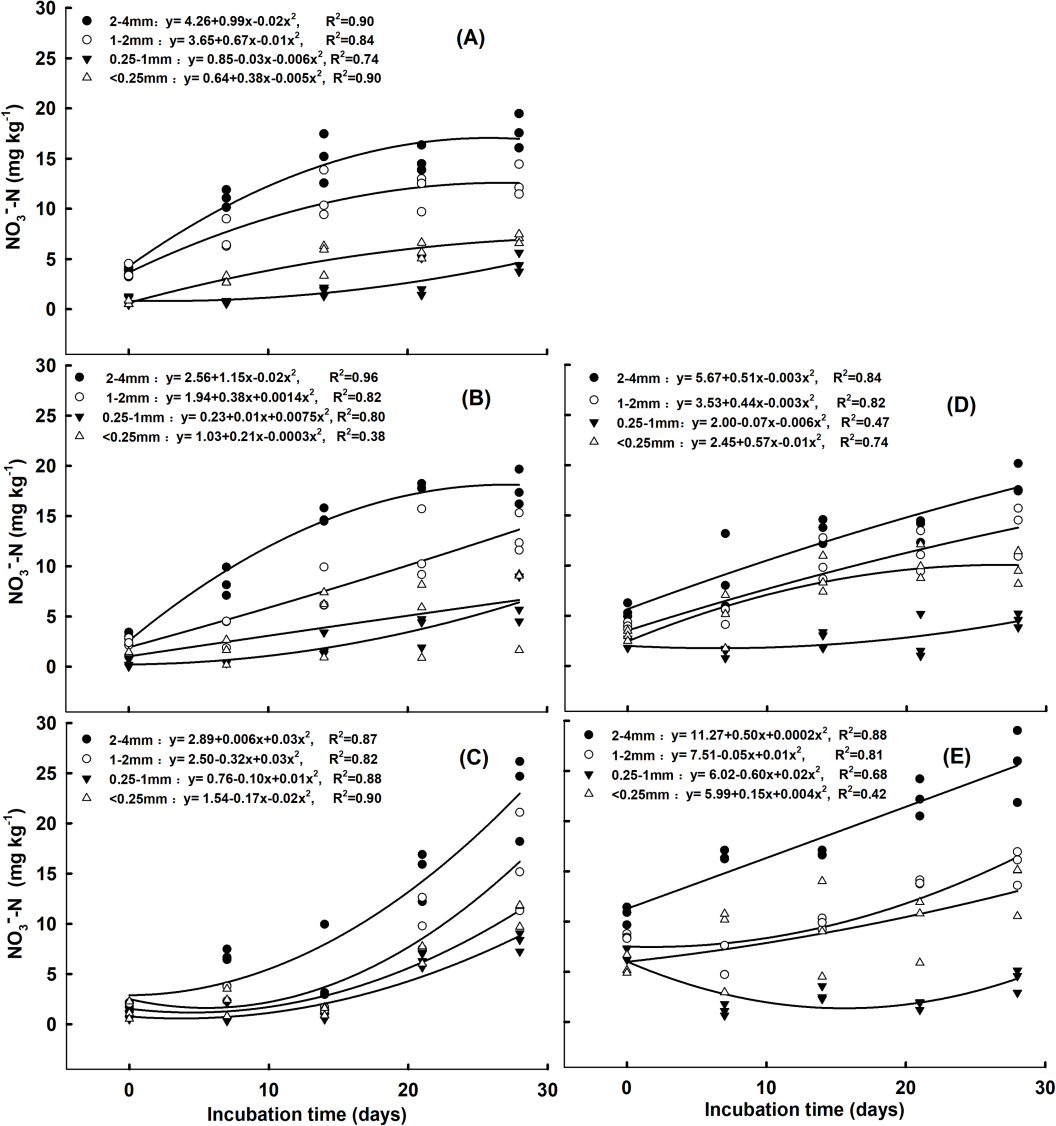
Correlation of NO_3_^−^-N concentrations with incubation time for the aggregate size classes in response to (A) no N addition, (B) addition of 5 mg NH_4_^+^-N kg^−1^ aggregate, (C) addition of 10 mg NH_4_^+^-N kg^−1^ aggregate, (D) addition of 5 mg NO_3_^−^-N kg^−1^ aggregate, and (E) addition of 10 mg NO_3_^−^-N kg^−1^ aggregate.

Under no N addition and the addition of 5 mg NH_4_^+^ kg^-1^ soil, as the incubation time increased, the NO_3_^−^-N concentration in the 2–4 mm, 1–2 mm and <0.25 mm aggregates increased, while it first decreased and then increased in the 0.25–1 mm aggregates (Figs. 1A and 1B). Following the addition of 10 mg NH_4_^+^ kg^−1^ soil, the NO_3_^−^-N concentration in the four soil aggregate size fractions increased as the incubation time increased (Fig. 1C). Under the addition of 5 mg and 10 mg NO_3_^−^ kg^−1^ soil, as the incubation time increased, the NO_3_^−^-N concentration in the 2–4 mm, 1–2 mm and <0.25 mm aggregates increased (Figs. 1D and 1E). For the 0.25–1 mm aggregates, the NO_3_^−^-N concentration first decreased, then increased under the addition of 5 mg NO_3_^−^ kg^−1^ soil. However, it decreased under the addition of 10 mg NO_3_^−^ kg^−1^ soil as the incubation time increased (Figs. 1D and 1E). The largest NO_3_^−^-N concentration was observed for the 2–4 mm aggregates, while the lowest was found in the 0.25–1 mm aggregates under both forms of N addition and rates (Figs. S1 and S2). Under the addition of 10 mg NH_4_^+^ kg^−1^ soil and 10 mg NO_3_^−^ kg^−1^ soil, the NO_3_^−^-N concentration in the 2–4 mm aggregates was significantly higher than that of the other aggregates on day 7, 14, 21, and 28 (Figs. S1B–S1E and S2B–S2E, *P* < 0.05). For the 2–4 mm aggregates, the NO_3_^−^-N concentration was significantly higher under the addition of 10 mg NO_3_^−^ kg^−1^ soil than the addition of 0 and 5 NO_3_^−^ kg^−1^ soil on day 7, 21, and 28 (Figs. S2B, S2D and S2E, *P* < 0.05).

### Response of N_r_ to soil aggregate sizes and N addition

**Figure 2:**
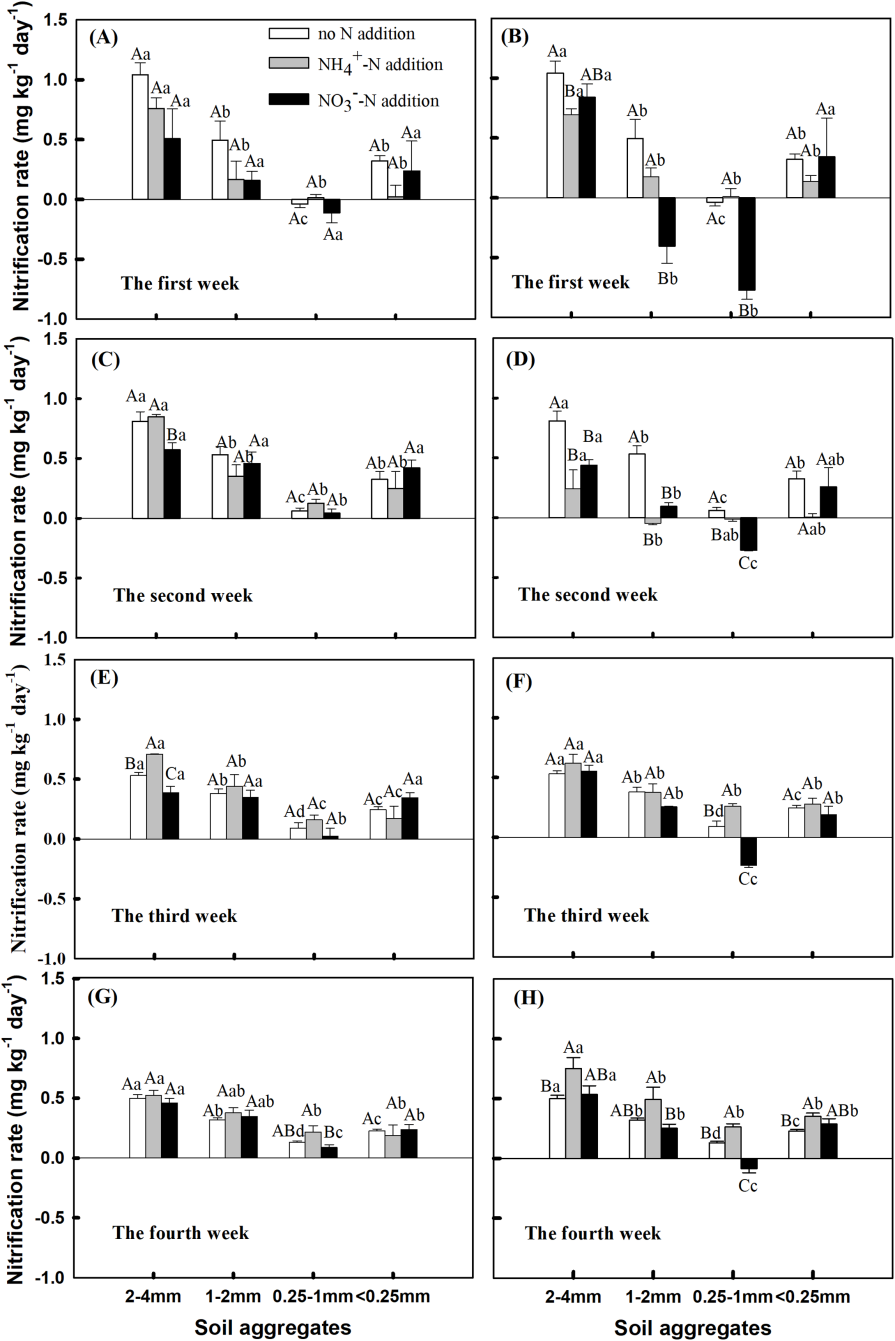
Effects of NH_4_^+^-N and NO_3_^−^-N addition on nitrification rate (N_r_) during the 4 incubation period among soil aggregates of different sizes (Mean ± SE, *n* = 3) under the (A, C, E and G) addition of 5 mg N kg^−1^ aggregate and (B, D, F and H) addition of 10 mg N kg^−1^ aggregate. Capital letters indicate significant differences among no N, NH_4_^+^-N, and NO_3_^−^-N treatments, and lowercase letters indicate significant differences among soil aggregate sizes (*P* < 0.05).

The rate of nitrification fluctuated greatly during the first and second weeks of incubation, especially in 0.25–1 mm and 1–2 mm aggregates (Figs. 2A–2D). After the 3rd week of incubation, the change of nitrification between aggregate sizes tended to be stable (Figs. 2E and 2F). During the 4 week incubation, nitrification rate in the 0.25–1 mm aggregates showed a consistent change with the addition of 10 mg NO_3_^−^ kg^−1^ soil, all of which were negative, but increased with the incubation time. Especially for the change in nitrification rate after 4 weeks of incubation, nitrification patterns associated with different sizes of aggregates were similar under the two N addition treatments, except for the 2–4 mm aggregates where NH_4_^+^ addition showed the highest N_r_ (Figs. 2G and 2H). The N_r_ value during the 4 week incubation period differed between aggregate sizes, with the 2–4 mm aggregates having significantly higher N_r_ than other aggregate sizes at the addition rate of 10 mg NH_4_^+^ kg^−1^ soil and 10 mg NO_3_^−^ kg^−1^ soil (Fig. 2H, *P* < 0.05). The largest N_r_ value (0.75 and 0.46 mg kg^−1^ day^−1^ for 10 mg NH_4_^+^ kg^−1^ soil NH_4_^+^ and 5 mg NO_3_^−^ kg^−1^ soil NO_3_^−^ addition, respectively) was observed for large macro-aggregates (2–4 mm), and the lowest for meso-aggregates (0.25–1 mm) (0.26 and −0.085 mg kg^−1^ day^−1^ for 10 mg NH_4_^+^ kg^−1^ soil NH_4_^+^ and 10 mg NO_3_^−^ kg^−1^ soil NO_3_^−^ addition, respectively). The N_r_ was significantly higher under the addition of 10 mg NH_4_^+^ kg^−1^ soil than in untreated soil, except for the 1–2 mm aggregates (Fig. 2H, *P* < 0.05). N_r_ was negative in the 0.25–1 mm aggregates under the addition of 10 mg NO_3_^−^ kg^−1^ soil.

The incubation period, soil aggregate size, nitrogen addition, and interactions of these variables had significant impacts on the N_r_ (Table S1, *P* = 0.005 and *P* < 0.0001). NH_4_^+^-N in soil aggregates was positively correlated with the soil N_r_, and the porosity of soil aggregates was positively correlated with the soil N_r_ under all N addition treatments (Table 2).

**Table 2:** Pearson correlation between the physiochemical properties of the soil aggregate sizes and nitrification rate across nitrogen (N) addition treatments.

N addition (mg N kg^-1^ soil)	Physiochemical properties of soil aggregates		
	SP	SOC	NH_4_^+^-N
No N addition	0.887**	0.209	0.864**
5 mg (NO_3_^−^-N)	0.778**	0.375	0.749**
10 mg (NO_3_^−^-N)	0.711**	0.398	0.706*
5 mg (NH_4_^+^-N)	0.795**	0.021	0.614*
10 mg (NH_4_^+^-N)	0.827**	0.084	0.695*
All of the treatments	0.717**	0.202	0.651**

**Notes:**

SP, soil porosity; SOC, soil organic carbon.

* *P* < 0.05.

** *P* < 0.01.

### Contribution of different types of aggregates to the N_r_ in bulk soil

**Figure 3:**
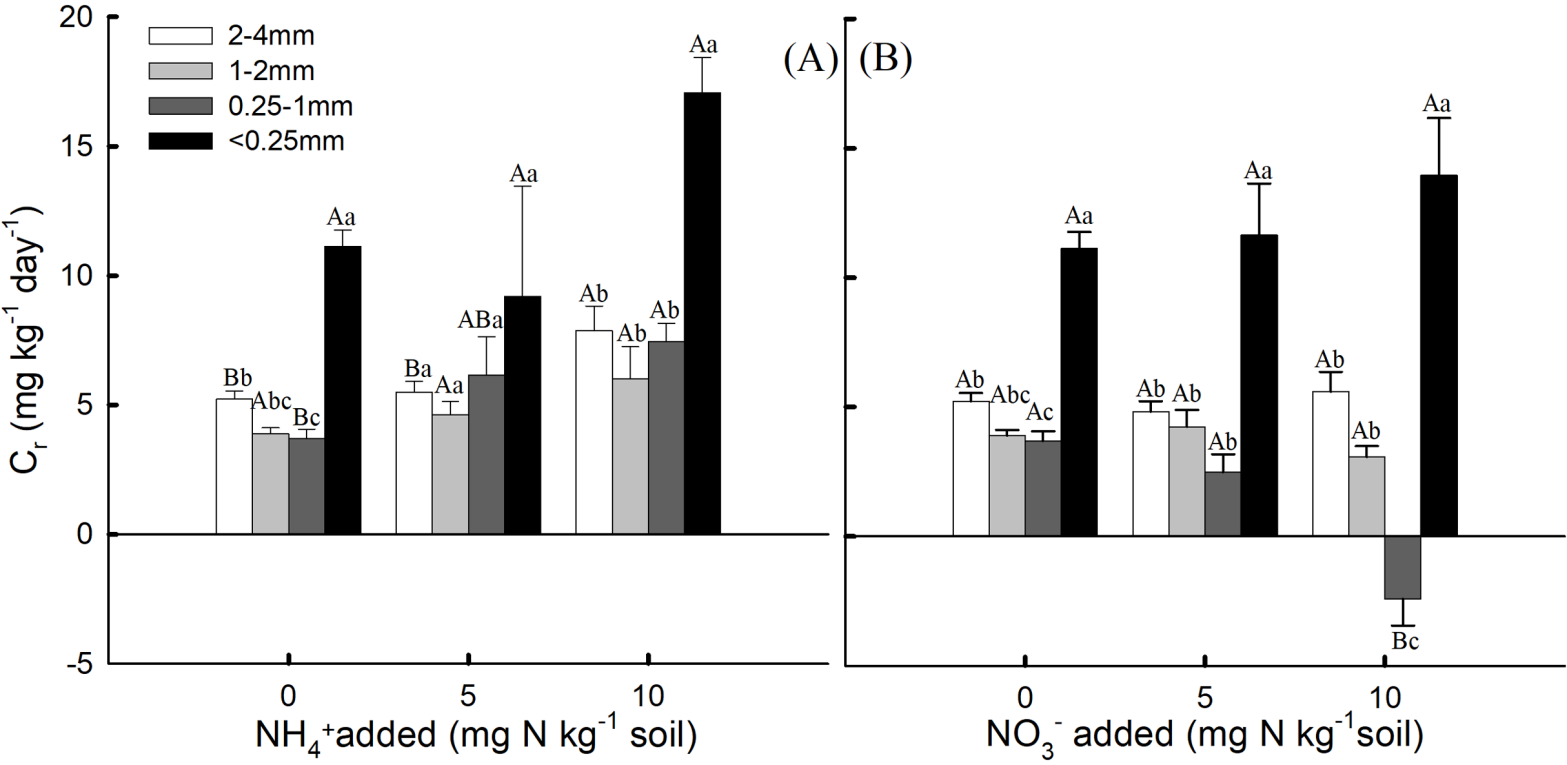
The contribution rate (C_r_) of nitrification rate (N_r_) for soil aggregates to the bulk soil under (A) NH_4_^+^-N addition and (B) NO_3_^−^-N addition treatments (Mean ± SE, *n* = 3) of the fourth week. Capital letters indicate significant differences among NH_4_^+^-N and NO_3_^−^-N concentrations and lowercase letters indicate significant differences among soil aggregate sizes (*P* < 0.05).

The proportion of the aggregate sizes in the bulk soil was 10.48, 12.21, 28.35 and 48.73% for the 2–4 mm, 1–2 mm, 0.25–1 mm, and <0.25 mm aggregates, respectively (Table 1). The <0.25 mm aggregates had a significantly higher proportion than the other aggregate sizes (*P* < 0.05). The 2–4 mm aggregates had higher soil N_r_, but the soil aggregate proportion for the 2–4 mm aggregates was low. Conversely, the 0.25–1 mm and <0.25 mm aggregates had lower soil N_r_, but their soil aggregates proportions were high. For the 2–4 mm aggregates, C_r_ was significantly higher with the addition of 10 mg NH_4_^+^ kg^−1^ soil than with the addition of 0 and 5 mg NH_4_^+^ kg^−1^ soil (Fig. 3A, *P* < 0.05). However, for the 0.25–1 mm aggregates, C_r_ was significantly lower with the addition of 10 mg NO_3_^−^ kg^−1^ soil than with the addition of 0 and 5 mg NO_3_^−^ kg^−1^ soil (Fig. 3B, *P* < 0.05).

## Discussion

### Variability of nitrification associated with soil aggregate sizes

Aggregate sizes had a significant effect on the nitrification rate (Jiang et al., 2011). In the present study, higher nitrification rates were found in 2–4 mm aggregates than in the other three aggregates under both N addition treatments. Muruganandam, Israel & Robarge (2010) found that a higher nitrification rate associated with 0.5–1 mm aggregates than 2–4 and <0.25 mm aggregates. In addition, Jiang et al. (2011) reported that nitrification rates were higher for the 2–0.25 mm fraction than the 0.25–0.053 mm fraction. We further separated the 0.25–2 mm aggregates into 0.25–1 and 1–2 mm aggregates and found higher nitrification rates in the 1–2 mm aggregates. However, a study of N turnover associated with aggregates in wetland soils revealed that the >2 mm aggregate fraction played a vital role in the release of inorganic N (Song et al., 2017), which was consistent with our results. Across different aggregate fractions, the heterogeneous distribution of microclimatic conditions and substrates of different qualities influence microbial community structure and nitrification rates (Bach & Hofmockel, 2014; Blaud et al., 2017; Davinic et al., 2012; Muruganandam, Israel & Robarge, 2010). Nitrification rates had no correlation with SOC. This may be due to the fact that the nitrification process was dominated by autotrophic microorganisms in our experiments. However, the NH_4_^+^-N in soil aggregates was positively correlated with the soil nitrification rate, which means that high concentration of NH_4_^+^ might improve the soil nitrification capacity (Zhao, Cai & Xu, 2007). In addition, there was a significant positive correlation between the porosity of soil aggregates and the nitrification rate, which may be a factor affecting the rate of nitrification. Specifically, a higher SP results in a higher specific surface area and therefore higher aerobic microbial biomass and activity. Oxygen availability is one of the most important factors controlling the rate of nitrification in soil (Princic et al., 1998). Aerobic microorganisms associated with nitrification are dependent on the presence of oxygen (Ke, Lu & Conrad, 2015). Because the rate of O_2_ diffusion is related to the aggregate radius, O_2_ concentrations are different in various aggregate sizes (Sexstone et al., 1985), and thus differentially affect the nitrification process. The 2–4 mm aggregates was the most active, which might be due to the high levels of SP allowing sufficient oxygen supply (Sexstone et al., 1985), and this in turn influences activities and compositions of soil microbial communities (Blackwood et al., 2006). In addition, our previous study has found that the abundance of bacteria in 2–4 mm and 1–2 mm aggregates were much higher than that in 0.25–1 mm and <0.25 mm aggregates (Yang, Liu & Zhang, 2019). We found that there was a significant positive correlation between nitrification rate and bacterial abundance of soil aggregates with different sizes under the treatment of 5 mg NH_4_^+^ addition kg^−1^ aggregate (Fig. S3). Our results suggest that the 2–4 mm and 1–2 mm aggregates could have a dominant influence on the response of soil N transformation processes to future nitrogen deposition in the studied alpine meadow.

### Relationship of nitrification rate with nitrogen addition

The nitrification rate of different aggregate fractions responded differently to two forms of N addition. Specifically, nitrification rate under the 10 mg NH_4_^+^ treatment was greater than that under the 10 mg NO_3_^−^ treatment across all soil aggregate fractions, which is in agreement with the results of previous studies (Jiang, Jin & Sun, 2014; Zhang et al., 2017). NH_4_^+^ concentration is important to the determination of ammonia oxidizer growth and whether oxidization will be conducted by bacteria or archaea (Verhamme, Prosser & Nicol, 2011). However, NO_3_^−^ is another primary inorganic N form in soils, and the impact of NO_3_^−^ on N_r_ is likely to be based on the product inhibition mechanism (Ying et al., 2017), which means that excessive concentration of the final product (NO_3_^−^) could inhibit the activity of nitrosating bacteria and nitrifying bacteria, thereby affecting soil nitrification capacity (Painter, 1977). In the present study, N_r_ was negative in the 0.25–1 mm aggregates when treated with 10 mg NO_3_^−^ kg^−1^ soil. This may have been caused by a product inhibition mechanism. On the one hand, the SP of the 0.25–1 mm aggregates was small and oxygen diffusion might be limited; therefore, the microorganisms in the aggregates may have been in an anoxic state (Ebrahimi & Or, 2016; Schlueter et al., 2018). On the other hand, a large amount of product was accumulated, which may have enabled denitrification (Su et al., 2019). As a result, the net nitrification rate of the 0.25–1 mm aggregates was negative under high NO_3_^−^-N addition. Deposition of NO_3_^−^-N has become increasingly important to the total N deposition (Zhao et al., 2009). Neumann et al. (2013) investigated the effects of fertilization on microbial communities in soil aggregates, and found that fertilizations increased the abundance of microbial communities in the larger-sized fractions than in fine silt. In our study, we found that, the soil nitrification rate in 2–4 mm aggregates was larger after N addition. Although we have not characterized the microbial community, our study also showed that the large aggregate size responds more strongly to fertilization. Blaud et al. (2017) found that different sieving methods affect the observed bacterial diversity and abundance. The dry sieving method used in our study can lead to different results as compared to the wet sieving method. Blaud et al. (2018) compared the abundance of N cycling genes in different land use and soil aggregates sizes, and found land use patterns had a significant impact on the abundances for all genes, while the effect of soil aggregates was relatively small. Our study found that there are differences in nitrification rates between different soil aggregates, and we suspect that their microbial distribution may also differ. The results of the present study showed that the soil nitrification in samples amended with 10 mg N kg^−1^ soil was greater than that of those treated with 5 mg N kg^−1^ soil. For the 0.25–1 mm aggregates, the NO_3_^−^-N concentrations in the 1st week were relatively stable or reduced in response to treatment with both forms of N, especially when 10 mg N kg^−1^ soil was added. These findings indicated that the nitrification ability differed among the soil aggregates of different sizes, which could be due to the SP and oxygen content. In macro-aggregates, the NO_3_^−^-N concentration increased linearly with the incubation time, indicating a stronger nitrification ability. In micro-aggregates, the NO_3_^−^-N concentration was exponentially related to the incubation time, presenting a stable or decreased nitrification ability.

### Contribution of aggregates to the nitrification rate in bulk soil

In this study, dry sieving revealed that the <0.25 mm aggregates was significantly higher than that of the other aggregates, representing 48.73% of the aggregate distribution. A previous study showed that soil fractions <0.25 mm represented only 2–20% of the aggregate distribution (Blaud et al., 2017). These findings indicated that different soils have different aggregate distributions, even when the same sieving method is used, so their contribution to nitrification will also differ. Fernández et al. (2010) compared the effects of no-till and conventional tillage on carbon contents and respiration rates for different aggregate size fractions, and they found that intermediate size aggregates showed the highest difference of C contents and the highest amount of respired C. This would indicate that C losses from soil through mineralization are mostly associated with intermediate aggregate size. Our previous research found that 0.25–1 and <0.25 mm aggregates had higher contribution rates to bulk soil SOC mineralization than 2–4 mm and 1–2 mm aggregates (Yang, Liu & Zhang, 2017). In our study, the 2–4 mm aggregates was the least represented, but had the highest nitrification rates. These findings imply that soil nitrification was mostly associated with the 2–4 mm aggregates. Almost half of the soil was composed of <0.25 mm aggregates, which resulted in higher contribution rates to the bulk soil nitrification rate than the other three aggregate sizes, despite the lower nitrification rate of the <0.25 mm aggregates.

## Conclusions

Nitrogen addition and soil aggregates had strong effects on nitrification rates. Moreover, the nitrification rate of the fourth week under high concentrations of NH_4_^+^ was higher than that of the NO_3_^−^ treated samples across all soil aggregate fractions. The higher nitrification capacity of the 2–4 mm aggregates could be explained by the maximum values of the SP (70.48%) likely allowing enough oxygen supply. Although soil nitrification was mostly associated with the 2–4 mm aggregates, half of the soil was composed of <0.25 mm aggregates (48.73%) in this study. These findings indicated that the <0.25 mm aggregates made a higher contribution to the nitrification rate in the bulk soil than the other fractions.

## Supplemental Information

10.7717/peerj.8230/supp-1 Supplemental Information 1 Raw data exported from flow-solution analyzer applied for data analyses and preparation for Fig. 1 and Figs. S1 and S2.

10.7717/peerj.8230/supp-2 Supplemental Information 2 Raw data of soil net nitrification rate calculated for each incubation period applied for data analyses and preparation for Fig. 2, Fig. S3 and Table S1.

10.7717/peerj.8230/supp-3 Supplemental Information 3 Raw data of contribution rate in the fourth week for data analyses and preparation for Fig. 3.

10.7717/peerj.8230/supp-4 Supplemental Information 4 Supplemental Figures and Tables.

## Additional Information and Declarations

### Data Availability

The following information was supplied regarding data availability: The raw data are available in the Supplemental Files.
