# Supplementary material for: Soil aggregate size influences the impact of inorganic nitrogen deposition on soil nitrification in an alpine meadow of the Qinghai–Tibet Plateau
Source: PeerJ. 2020 Jan 7;8:e8230. doi: 10.7717/peerj.8230 (PMC6953337; doi:10.7717/peerj.8230)
Supplement: Supplemental Information 4 [file peerj-08-8230-s004.docx]

**Soil aggregate size influences the impact of** **inorganic nitrogen deposition on soil** **nitrification in an alpine meadow of the Qinghai-Tibet Plateau**

Jingjing Li^1^, Chao Yang^2^, Xiaoli Liu^1^, Hanzhong Ji^3^ and Xinqing Shao^1,4,5*^

^1^ College of Grassland Science and Technology, China Agricultural University, Beijing, China.

^2^ Grassland Agri-Husbandry Research Center, College of Grassland Science, Qingdao Agricultural University, Qingdao, China

^3^ Institute of Haibei Tibetan Autonomous Prefecture Animal Husbandry and Veterinary Science, Xining, China.

^4^ Technical Platform for Adaptive Management of Livestock System in Alpine Grassland, Xining, China.

^5^ Key Laboratory of Restoration Ecology of Cold Area in Qinghai province, Northwest Institute of Plateau Biology, Chinese Academy of Sciences, Xining, China.

^*^Corresponding Author:

Xinqing Shao

Email address: shaoxinqing@163.com


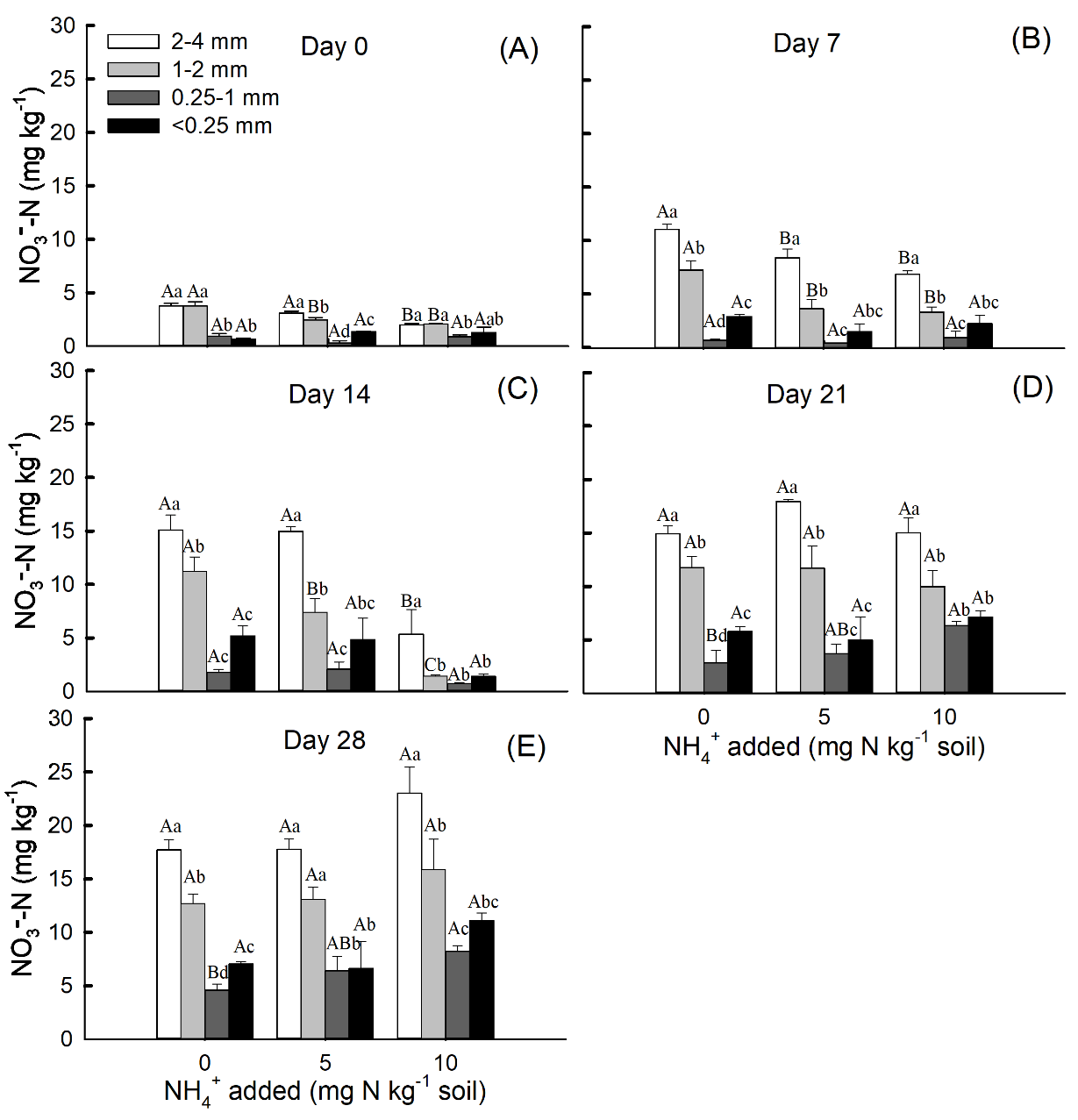


Figure S1. Effects of soil aggregate sizes on NO_3_^-^-N concentrations (Mean ± SE, n=3) under NH_4_^+^-N addition treatment (A, day 0; B, day 7; C, day 14; D, day 21; E, day 28). Capital letters indicate significant differences among different NH_4_^+^-N concentrations for the same soil aggregate size on 5 sampling dates, and lowercase letters indicate significant differences among different soil aggregate sizes under the same NH_4_^+^-N concentration on 5 sampling dates (*P* < 0.05).


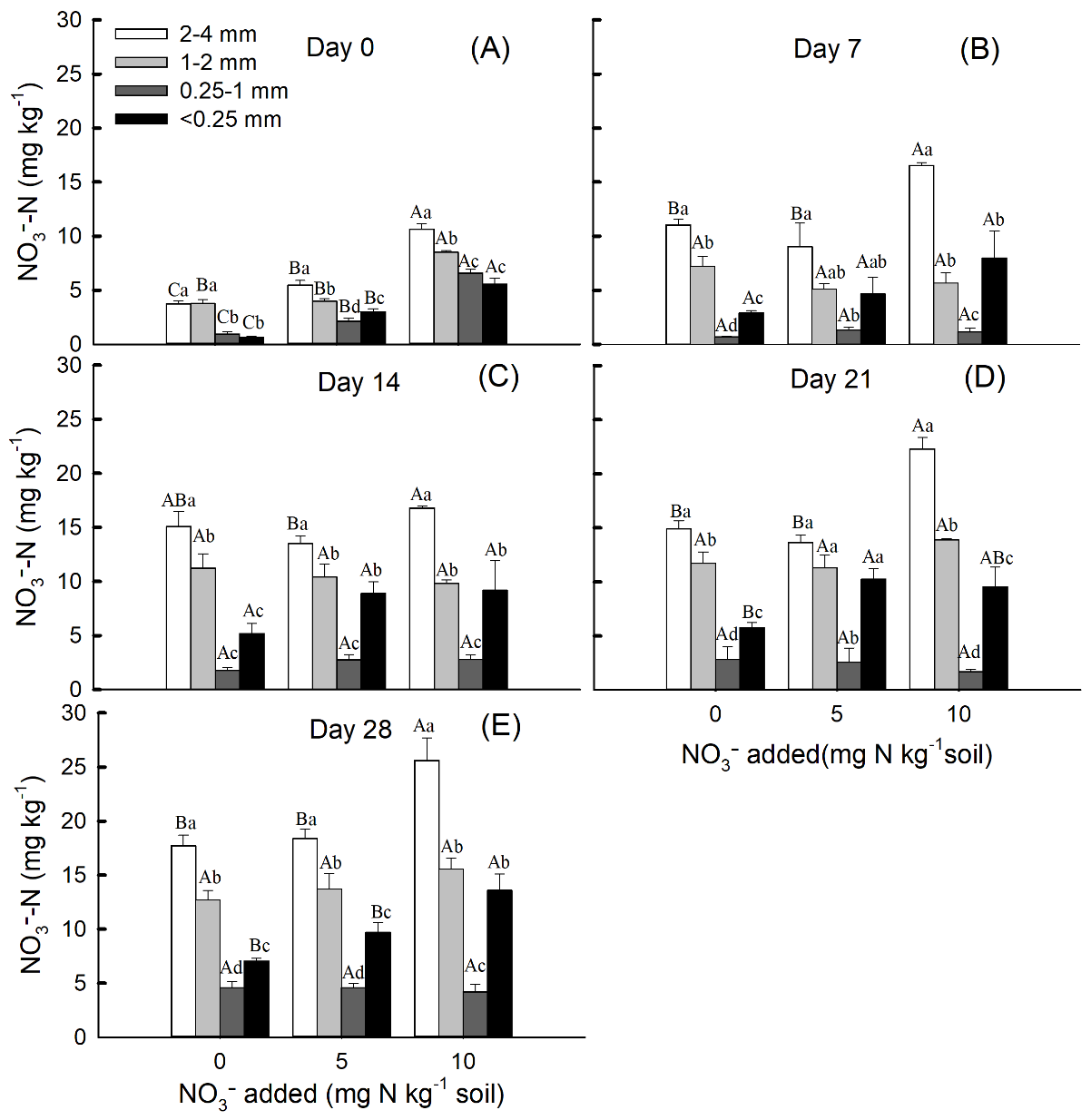


Figure S2. Effects of soil aggregate sizes on NO_3_^-^-N concentrations (Mean ± SE, n=3) under NO_3_^-^-N addition treatment (A, day 0; B, day 7; C, day 14; D, day 21; E, day 28). Capital letters indicate significant differences among different NO_3_^-^-N concentrations for the same soil aggregate size on 5 sampling dates, and lowercase letters indicate significant differences among different soil aggregate sizes under the same NO_3_^-^-N concentration on 5 sampling dates (*P* < 0.05).


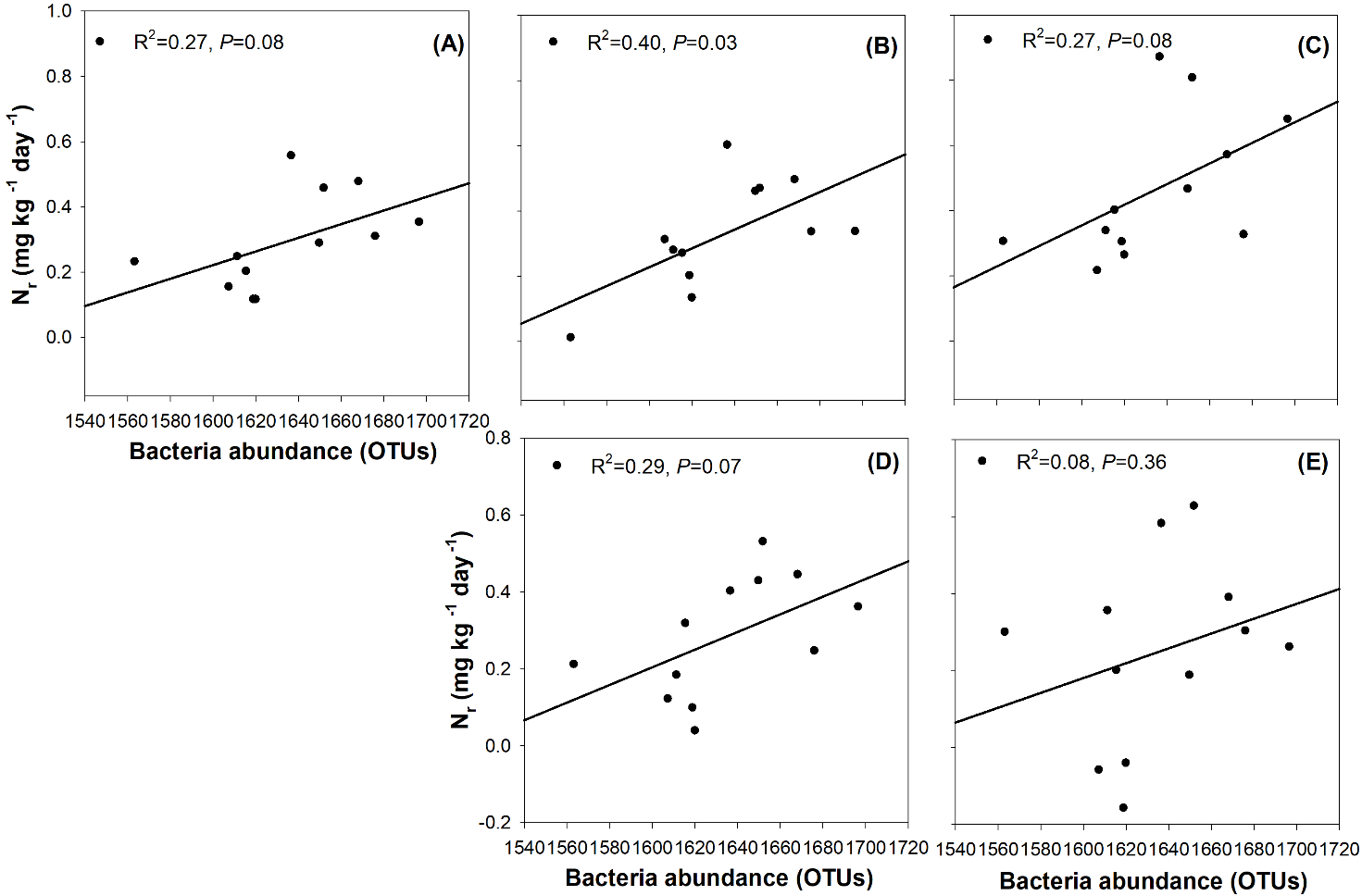


Figure S3. Correlations between the nitrification rate (N_r_) in the fourth week and bacteria abundance (OTUs) of soil aggregate sizes. (A) no N addition, (B) addition of 5 mg NH_4_^+^-N kg^-1^ aggregate, (C) addition of 10 mg NH_4_^+^-N kg^-1^ aggregate, (D) addition of 5 mg NO_3_^-^-N kg^-1^ aggregate, and (E) addition of 10 mg NO_3_^-^-N kg^-1^ aggregate.

Table S1. The three-way ANOVA results for evaluating the 3 main factors (incubation period, soil aggregate size, and nitrogen addition) influencing the nitrification rate (N_r_).

| Source | N_r_ (mg kg^-1^ day^-1^) | | |  |
| --- | --- | --- | --- | --- |
|  | df | F | P | |
| Incubation period | 3 | 4.453 | 0.005 | |
| Aggregate size | 3 | 170.229 | < 0.0001 | |
| Nitrogen addition | 4 | 15.963 | < 0.0001 | |
| Incubation period * Aggregate size | 9 | 7.497 | < 0.0001 | |
| Incubation period * Nitrogen addition | 12 | 6.892 | < 0.0001 | |
| Aggregate size * Nitrogen addition | 12 | 7.478 | < 0.0001 | |
| Incubation period *Aggregate size *Nitrogen addition | 36 | 1.448 | 0.064 | |
